# Orthostatic variation of pulmonary artery pressure in ambulatory heart failure patients

**DOI:** 10.1186/s12872-023-03534-y

**Published:** 2023-10-10

**Authors:** Prince Sethi, Prakash Acharya, Payton Lancaster, Brianna Stack, Kartik Munshi, Sagar Ranka, Zubair Shah, Andrew J. Sauer, Kamal Gupta

**Affiliations:** Department of Cardiovascular Disease, University of Kansas Medical Center, 3901 Rainbow Blvd, Kansas City, KS 66160 USA

**Keywords:** Orthostatic variation, Pulmonary artery pressure, CardioMEMS, Heart failure

## Abstract

**Aim:**

To study effect of change in position (supine and standing) on pulmonary artery pressure (PAP) in ambulatory heart failure (HF) patients.

**Methods:**

Seventeen patients with CardioMEMS® sensor and stable heart failure were consented and included in this single center study. Supine and standing measurements were obtained with at least 5 min interval between the two positions. These measurements included PAP readings utilizing the manufacturer handheld interrogator obtaining 10 s data in addition to the systemic blood pressure and heart rate recordings.

**Results:**

Mean supine and standing readings and their difference (Δ) were as follows respectively: Systolic PAP were 33.4 (± 11.19), 23.6 (± 10) and Δ was 9.9 mmHg (p = 0.0001), diastolic PAP were 14.2 (± 5.6), 7.9 (± 5.7) and Δ was 6.3 mmHg (p = 0.0001) and mean PAP were 21.8 (± 7.8), 14 (± 7.2) and Δ was 7.4 mmHg (p = 0.0001) while the systemic blood pressure did not vary significantly.

**Conclusion:**

There is orthostatic variation of PAP in ambulatory HF patients demonstrating a mean decline with standing in diastolic PAP by 6.3 mmHg, systolic PAP by 9.9 mmHg and mean PAP by 7.4 mmHg in absence of significant orthostatic variation in systemic blood pressure or heart rate. These findings have significant clinical implications and inform that PAP in each patient should always be measured in the same position. Since initial readings at the time of implant were taken in supine position, it may be best to use supine position or to obtain a baseline standing PAP reading if standing PAP is planned on being used.

## Background

Heart failure (HF) is currently a major health care burden in the United States and in 2012 it costed nation ~$37 billion [1]. Central to HF syndrome is the development of perfusion mismatch compared to peripheral tissue demand. This leads to the development of peripheral vasoconstriction, left ventricular hypertrophy and pulmonary hypertension as a compensatory mechanism. Typically, pulmonary congestion has been used for assessment of severity of left sided HF (reduced and/or preserved left ventricular function) [1, 2]. However, there has been increasing consideration of elevated pulmonary artery pressure as a contributor towards left heart failure and not just a consequent symptom, eventually reflecting worse prognosis. One such hypothesis involves pulmonary hypertension leading to right ventricular dysfunction and subsequently leading to left ventricular dysfunction. These have led to management of HF syndrome with vasodilators to improve peripheral perfusion and diuretics to reduce the volume overload/pulmonary congestion for symptomatic treatment.

This has further led to emphasis on the study of systemic and pulmonary circulation. There is better understanding of physiological and pathological changes in systemic circulation than pulmonary circulation due to relatively easy access of systemic peripheral vasculature compared to central pulmonary vasculature. For example, orthostatic and diurnal variation and their pathological association has been better studied for systemic circulation by means of invasive and non-invasive studies. Night-time systemic BP is typically lower and non-reduction/dipping or elevated night-time BP are associated with HF development [3]. Similarly, there is orthostatic reduction of systolic BP (~ 5–10 mmHg) with small increase in heart rate (10-20 bpm) on standing. These changes are predominantly attributed to pooling of blood in peripheral venous system leading to reduction in ventricular filling and subsequent reduction in cardiac output on standing and reduction in sympathetic tone at night-time [4–6].

Pulmonary circulation has more limited literature for the physiological and pathological variations and subsequent clinical impact of these variations. This limitation has been predominantly due to the need for invasive evaluation of pulmonary vasculature which has central intrathoracic location. Limited literature on pulmonary artery pressure (PAP) has shown that there is diurnal as well as orthostatic variation of PAP. However, due to the indwelling nature of catheters used to measure PAP and often studies involving subjects with primary pulmonary hypertension, the data may not translate to left sided HF population.

This challenge in measurement of PAP has been recently overcome with the commercial availability of implantable PAP monitoring system (CardioMEMS® system) for remote monitoring in HF. The utilization of ambulatory PAP in HF management as well as for research purposes has increased significantly. Sethi et al. recently utilized this technology to provide insights into the diurnal variation of PAP in stable ambulatory HF patients where the night-time systolic and mean PAP were elevated at night-time (2.6 and 1.2 mmHg respectively) [7–10].

The PAP is higher in supine position than upright position. This is thought to be due to increased venous return in supine position leading to increased pulmonary capillary wedge pressure and PAP without affecting pulmonary vascular resistance. A healthy variation is not well known but a range of 1–7 mmHg mean PAP decrease with upright position has been reported. There is further paucity of data on orthostatic variation of PAP in ambulatory HF patients. Raeside et al. studied six subjects with pulmonary hypertension with connective tissue disease with use of an invasive micromanometer tipped catheter. The investigators found that the standing PAP were lower than the supine position [11]. Berlier et al. studied supine and upright (45-degree elevation) PAP at rest (self-controlled) and exercise (historical control cohort) utilizing indwelling catheter. They studied 483 patients with a wide variety of indications but only 9–11% patients with post capillary pulmonary hypertension. They demonstrated ~ 1 mmHg lower upright mean PAP at rest and ~ 7 mmHg lower upright PAP at exercise end [12]. Mizumi et al. invasively evaluated upright and supine PAP in 17 patients with previously treated chronic thromboembolic pulmonary hypertension and showed ~ 5 mmHg elevation of mean PAP in upright position [13].

With the increasing use of ambulatory PAP both for clinical and research uses, it becomes important to understand the orthostatic variation in PAP in ambulatory patients as it has direct clinical implication in management of HF patients.

### Aim

The aim of this research was to study the effect of change in position (supine and standing) on PAP in ambulatory HF patients.

## Methods

We conducted a single center prospective study at large university setting, tertiary care hospital with advanced heart failure services. This was approved by the institutional review board and human subjects committee.

The inclusion criteria included >/= 18-year-old ambulatory HF patients with previously implanted CardioMEMS® sensor who had stable HF at the time of enrollment from heart failure clinic and no diuretic medication change in preceding 2 weeks. The exclusion criteria included patient’s unwillingness or inability to obtain PAP and systemic BP and heart rate in supine and standing positions. Informed consent as approved by the IRB was obtained from all patients enrolled in the study (Table 1).

**Table 1:** Inclusion and exclusion criteria

Inclusion criteria	Exclusion criteria
>/= 18-year-old ambulatory heart failure patients with previously implanted CardioMEMS sensor	Unwillingness or inability to obtain PAP and systemic BP and heart rate in supine and standing positions
Stable HF without diuretic medication change in preceding 2 weeks	

Subjects were selected from heart failure clinic during clinic hours from 8AM to 5 PM and measurements were obtained at their clinic visit. No preceding activity or other restrictions were applied to subjects. All subjects had CMS 3000 CardioMEMS sensor. The enrollment was performed from December 2020 through January 2022. This enrollment period coincided with COVID-19 pandemic and higher proportion of HF patients were managed remotely limiting the sample size. The data was successfully obtained in all enrolled subjects. Supine measurements were obtained in all subjects. Subsequently, patients were made to stand up and with at least 5 min interval between the two positions, measurements were obtained in an upright/standing position. These measurements included PAP readings utilizing the manufacturer handheld equipment (Model: CMS 3000) obtaining 10 s data in addition to the non-invasive systemic blood pressure and heart rate recordings. The PAP data was screened for noise and a single set of noise-free readings were recorded during each measurement at the discretion of the examiner.

Categorical and continuous variables are reported as percentages and mean +/- SD respectively. The data was tested for normality with Shapiro-Wilk test which showed that data followed a normal distribution (Table 2). A paired T-test was used to compare the supine and standing PAP values. A p-value of < 0.05 was considered statistically significant.

**Table 2:** Shapiro-Wilk test showing normal data distribution. PAP: Pulmonary artery pressure, SBP: Systolic blood pressure, DBP: Diastolic blood pressure

Difference between supine and standing data	Shapiro-Wilk test - p-value
Systolic PAP	0.2674
Diastolic PAP	0.5299
Mean PAP	0.8680
Systemic SBP	0.1800
Systemic DBP	0.1207
Heart rate	0.0519

## Results

Seventeen subjects completed the study procedures from December 2020 through January 2022 and were included in the study analysis. Mean age was 76.2 (± 6.3) years, 35.2% were females, 94.1% were Caucasians, mean BMI was 34.9 kg/sq.m, 47.1% had coronary artery disease, 94.1% had hypertension, 64.7% had diabetes mellitus, 70.6% had obstructive sleep apnea, 35.3% had chronic obstructive pulmonary disease and 70.6% had chronic kidney disease. 47.1% had HF with reduced ejection fraction (LVEF < 40%), 11.7% had HF with recovered LVEF and remaining HF with preserved LVEF. A specific medication list was not recorded in this study.

**Fig. 1:**
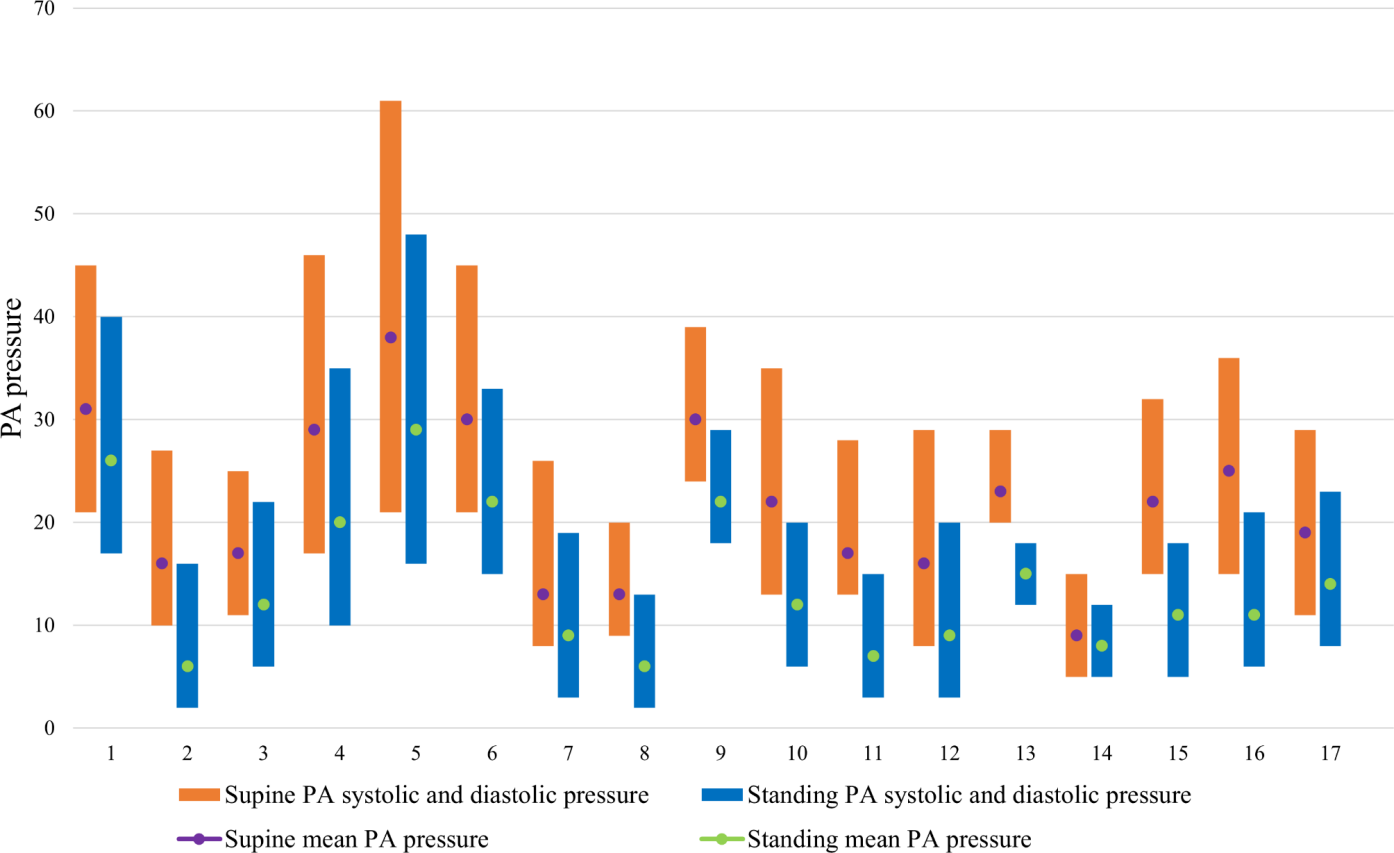
Variation of pulmonary artery pressure (in mmHg) between standing and supine positions for individual subjects (1–17)

There was a significant decrease in systolic PAP, diastolic PAP and mean PAP in the standing position as compared to the supine position (Table 3; Fig. 1) in the overall group. There was no significant change in the systemic BP or heart rate.

**Table 3:** Variation of mean pulmonary artery pressure (PAP), systemic blood pressure and heart rate between standing and supine positions

	Supine	Standing	p-value
Systolic PAP, mean (SD) in mmHg	33.4 (11.19)	23.5 (10.1)	0.0001
Diastolic PAP, mean (SD) in mmHg	14.2 (5.6)	7.9 (5.7)	0.0001
Mean PAP, mean (SD) in mmHg	21.8 (7.8)	14 (7.2)	0.0001
Systemic SBP, mean (SD) in mmHg	124.4 (10.17)	125.9 (14.4)	0.734
Systemic DBP, mean (SD) in mmHg	70.4 (11.5)	68.5 (8.3)	0.3336
Heart rate, mean (SD) In bpm	75.6 (13.2)	79.1 (15.7)	0.086

## Discussion

PAP has been increasingly shown to play a central role in the management of ambulatory HF patients. Due to the inherent central intrathoracic location of the pulmonary vasculature, there has been lack of literature towards understanding pathophysiological PAP variations which is further limited in ambulatory setting. The available studies have predominantly utilized indwelling catheters which may not apply to the ambulatory patient population. There has been some data showing the diurnal PAP variation in the ambulatory setting but there is no data to date about orthostatic ambulatory PAP variation. This is the first study to our knowledge evaluating the effect of body positional change in ambulatory HF patients.

Our study shows reduction in upright PAP (~ 8 mmHg mean PAP reduction) which is consistent with studies conducted with indwelling catheters (showing wide range of variation 1–7 mmHg). This has been thought to occur due to reduction in venous return to right and left heart and subsequent post capillary PAP reduction without affecting the pulmonary vascular resistance. Our patient population did not show significant variation in systemic BP or heart rate and this is likely due to > 5 min interval between the supine and standing measurements allowing time for systemic compensation with increase in systemic vascular resistance.

This study’s limitations include small sample size, predominant caucasian population (94%), male population (65%) and being limited to compensated HF patients on stable dose of diuretic. This study also did not evaluate the effect of comorbidities, gender and specific medications (or dosages). Thus, we are unable to say if these findings would also be seen in those not on diuretics or in a decompensated state.

## Conclusion

These findings have significant clinical implications and inform that PAP in each patient should always be measured in the same position. Since initial readings at the time of implant are taken in supine position, it may be best to use supine position or to obtain a baseline standing PAP reading if standing PAP is planned on being used. A larger study should be considered in the future for findings which could be applicable to larger population.

## Data Availability

The datasets used and/or analyzed during the current study are not publicly available due to privacy concerns but are available from the corresponding author [K.G.] upon reasonable request.

## Abbreviations

PAP: Pulmonary artery pressure
PA: Pulmonary artery
SBP: Systolic blood pressure
DBP: Diastolic blood pressure
SD: Standard deviation
